# Effect of Enzymatic Depolymerization of Cellulose and Hemicelluloses on the Direct Dissolution of Prehydrolysis Kraft Dissolving Pulp

**DOI:** 10.1021/acs.biomac.1c01102

**Published:** 2021-10-21

**Authors:** Sara Ceccherini, Marina Ståhl, Daisuke Sawada, Michael Hummel, Thaddeus C. Maloney

**Affiliations:** † Department of Bioproducts and Biosystems, Aalto University, P.O. Box 16300, FI-00076 Aalto, Finland; ‡ Stora Enso, Imatra Research Center, Tornansaarenraitti 48, Imatra FI-55400, Finland

## Abstract

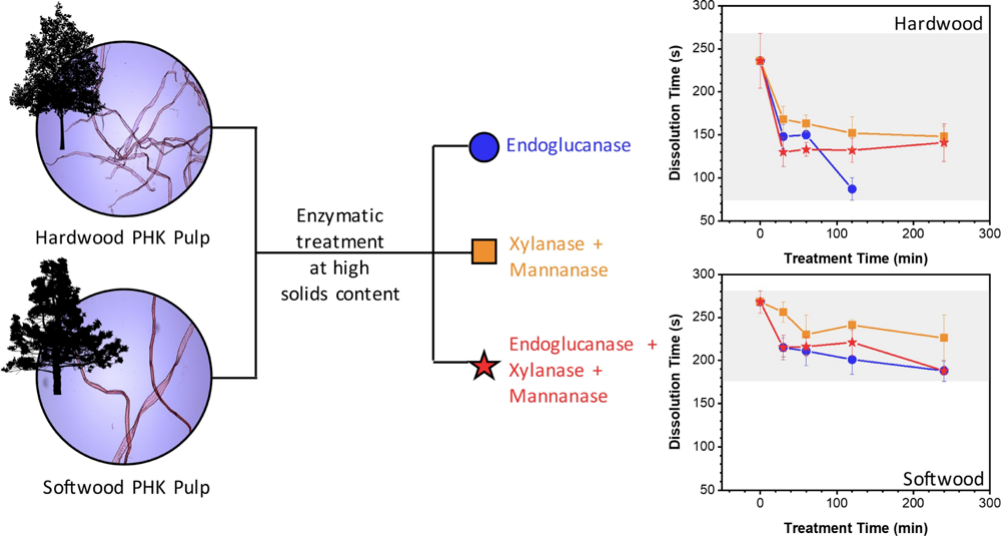

Prehydrolysis kraft (PHK) pulps account for more than half of the global market of dissolving pulp. Characterized by high reactivity toward dissolution, their performances can still be improved by activation treatments. This study compares the dissolution kinetics in cupriethylenediamine of a hardwood and a softwood PHK pulps before and after their activation by high-solid-content mechano-enzymatic treatments. Three enzyme combinations were tested: endoglucanase (E), xylanase and mannanase (XM), and endoglucanase, xylanase, and mannanase (EXM). Xylanase and mannanase reduced the hemicellulose content of only hardwood (by max. 2.4%). Mixing and carbohydrate depolymerization decreased the dissolution time of hardwood and softwood pulps by a maximum of 63 and 30% with E, 37 and 16% with XM, and 44 and 30% with EXM, respectively. The shortening of the dissolution time was partially hindered by hornification, which increased with hemicellulose degradation. Interestingly, XM accelerated the dissolution while preserving a high weight-average molecular mass.

## 1 Introduction

Dissolving pulp is characterized by high cellulose purity (>92%), high brightness, low macromolecular polydispersity, low ash and metal ion content, and remarkable reactivity.^1,2^ Principally, it is used to produce cellulose derivative products, such as textile fibers, acetate filaments and films, binders, detergents, food and pharmaceutical additives, explosives, and specialty papers.^3,4^ Different final products require different pulp chemistries and processes, such as xanthation and regeneration in the case of viscose fibers or acetylation in the case of acetate filaments or films.^1,4^ Therefore, the reactivity of dissolving pulp is evaluated in the context of its final application.

Due to the increasing demand for cellulose derivative products, the global production of dissolving pulp has increased from 5.6 MMT in 2013 to a nominal capacity of 10.5 MMT in 2020.^4,5^ This market consists mainly of acid sulfite (AS) and prehydrolysis kraft (PHK) dissolving pulp. In recent years, the interest in prehydrolysis kraft pulp has grown as market development in paper pulp has largely stagnated. In 2003, the global market of dissolving pulp consisted of 60–63% acid sulfite (AS) pulp, 22–25% prehydrolysis kraft (PHK) pulp, and 12–16% cotton linter pulp,^1^ while at present, it is composed of ca. 56% PHK pulp and 42% AS pulp.^2^

Prehydrolysis kraft pulping is a multistage process. Traditional kraft pulping is not capable of selectively removing short-chain hemicelluloses because, during the process, hemicelluloses become increasingly resistant to alkaline degradation. Therefore, a steam- or water-based pretreatment promoting acid hydrolytic degradation—the prehydrolysis—is performed before alkaline kraft pulping to reach the desired low hemicellulose content. Kraft pulping can reach a maximum of ca. 86% alpha-cellulose, while dissolving pulp generally requires a minimum of 92%.^1^ The quality and the yield of PHK pulp depend on the raw material, the prehydrolysis conditions, and the kraft cooking conditions.^1,6^

When PHK pulps are used to produce man-made cellulose fibers, their quality is strongly related to their reactivity toward dissolution, which can be interpreted as the extent or the ease at which fibers dissolve. It is generally acknowledged that pulp reactivity depends on pulp purity and the molecular mass distribution. Even if these features can be tailored according to the desired end products, the molecular mass distribution of PHK pulps is usually rather uniform^7,8^ and has a polydispersity index (PDI) around 3–4.5.^7,9^ Lignin- and hemicellulose-based impurities tend to measure around 0.05 and 2–8%, respectively.^1^

Hemicelluloses are notoriously noxious for the manufacturing of man-made cellulose fibers. For example, in viscose production, they consume oxygen hampering cellulose degradation during aging, react preferentially with CS_2_ during xanthation causing inhomogeneities, and weaken and decolorize the final fibers.^1^ After PHK pulping, regardless of the wood species, hemicelluloses are mainly xylan and glucomannan.^10,11^ Both xylans and glucomannans are mostly linear polymers as side chain substituents such as the acetyl groups, galactose, arabinose, rhamnose, and galacturonic acid are almost completely removed during prehydrolysis and pulping.^1,11^ Moreover, until their cleavage, some side chains tend to hamper the peeling reaction of hemicellulose backbones. It follows that part of PHK hemicelluloses has high molecular weight, low branching, and limited content of uronic side chains. All these characteristics make these hemicelluloses inclined to co-crystallize on cellulose and to become alkali resistant.^7,9,11,12^

The reactivity of PHK can be further improved by mechanical, chemical, and enzymatic treatments. When the treatment is successful, the pulp is *activated* and, for instance, it can dissolve faster and/or more thoroughly than before the treatment. Enzymatic treatments on pulps at both low and high solid contents have been widely reported as promising activation approaches. However, high solid contents can achieve more successful activations. These are the results of a mild refining action caused by the friction between the fibers (e.g. increase in surface fibrillation and porosity) and of the improved physical association between the enzymes and their substrate.^13,14^

Endoglucanases are among the enzymes that have been proven to be most successful in activating wood pulp.^15−17^ Belonging to cellulases, their activation relies on cellulose hydrolysis, which in turn affects several pulp properties. Pulp intrinsic viscosity decreases, fibers become shorter, and fines, porosity, and fibrillation increase. Considering that shorter polymeric chains are faster to dissolve^18^ and that fibers with a higher surface area are more accessible to solvents,^19^ endoglucanase can improve pulp dissolution. At the same time, depolymerization must be limited to prevent an excessive decrease of the mechanical properties of the regenerated cellulose fibers.^20,21^

Hemicellulases are another class of enzymes that can increase pulp reactivity. Xylanases and mannanases belong to this class, and they depolymerize xylans and glucomannans, respectively. If hemicelluloses are removed and pulp purity is increased, cellulose becomes more accessible to reagents and eventually to other enzymes.^22−24^ For instance, xylanase and endoglucanases have been applied together to upgrade kraft pulp to dissolving pulp.^22,23^ In these studies, the two enzymes acted synergistically, depolymerized xylan and cellulose more effectively than when used individually, and the treated pulps reached more thorough dissolutions.

The accessibility of cellulases and hemicellulases to the carbohydrates of wood pulp depends on the molecular size and the structure of the enzymes and on several pulp features, including specific surface area, porosity, supramolecular organization, and the covalent linkages within the lignin-carbohydrate complexes.^25^ Therefore, the synergy between hemicellulases and endoglucanase that has been observed using paper-grade pulps may not necessarily occur using high-purity dissolving pulps.^26^ In the case of dissolving pulps, the activation could be hindered both by the different chemistry, distribution, and content of the hemicelluloses and by the lower average pore size of pulp fibers. During the manufacturing of pulps with very low yield such as dissolving pulps, the microfibrils tend to aggregate and, consequently, pulp porosity is reduced.^27^

While previous studies verified that endoglucanases and hemicellulases could enhance pulp dissolution extent,^15,23,26^ the present study investigates whether the same enzymes could accelerate the direct dissolution of softwood and hardwood never-dried PHK pulps. Three treatments were tested: the first used only the endoglucanase, the second used xylanase and mannanase, and the third combined endoglucanase, xylanase, and mannanase. Each treatment was performed using the pulp at high solid content. It was hypothesized that the depolymerization of xylan and glucomannan could have helped in increasing the accessibility of endoglucanase to cellulose and that the pulps treated with both endoglucanase and hemicellulases would have dissolved faster than those treated with endoglucanase alone.

## 2 Materials and Methods

### 2.1 Samples

The pulps used for this study were never-dried hardwood (mixture of *Betula* sp.—ca. 95%–and *Populus* sp.—ca. 5% -) and never-dried softwood (mixture of *Picea abies* and *Pinus sylvestris*) prehydrolysis kraft dissolving pulps from a Finnish mill.

The mechano-enzymatic treatments were performed with three commercial enzyme mixtures from AB Enzymes: Ecopulp R, Ecopulp TX800A, and EL-2019/003343. The main activity of the mixtures was endoglucanase, xylanase, and mannanase, respectively. The treatments were labeled as follows: *E treatments* when using only endoglucanase, *XM treatments* when using mixed xylanase and mannanase, and *EXM treatments* when using mixed endoglucanase, xylanase, and mannanase.

Prior to each treatment, the pulp was adjusted at ca. 25% (w/w) solid content. First, the pH was set at 6.5, and then, the pulp was transferred into a Kenwood mixer and heated up to 55 °C under constant mixing (speed 1). The pulp temperature remained constant until the end of the treatment. The dosage of each enzyme was 0.2 mg enzyme product/g (oven-dry basis) of the pulp, also when the enzymes were used together. The enzymes were first diluted with deionized water and then sprayed manually onto the mixing pulp. In XM and EXM treatments, the commercial mixtures were mixed prior to dilution. The volume of water used for the dilution was equivalent to the water necessary to lower the pulp solid content to 20% (w/w). Each treatment was run four times using batches of 100 g (oven-dry basis) of pulp. At the designated times, an amount of ca. 25 g (oven-dry basis) of pulp was collected and deactivated. To deactivate the enzymes, pulps were washed first with boiling and then with room temperature deionized water. Finally, the four small portions were mixed to form one sample, closed in a plastic bag, and cooled in an ice bath for minimum 2 h. The chosen treatment times were 30, 60, 120, and 240 min.

### 2.2 Characterization Analyses

#### 2.2.1 Fiber Morphology and Water Retention Value

Fiber length, fines content, fibrillation, and cell wall thickness were measured with a METSO Fiber Lab SN Analyzer. The results were compared with the analysis of the samples by optical microscope (Leica DM750). Prior to imaging, pulp fibers were adjusted to ca. 1.5 wt % solid content and 10 mL of pulp suspension was dyed using 1 mL of aqueous safranine solution at 1 wt %. Pulp porosity (fiber saturation point, FSP) and pulp micropores were assessed by the two-point solute exclusion technique as described in the literature.^28^ Fiber pores were classified into micropores (<3.2 nm) and mesopores (3.2–54 nm). The water retention value (WRV) was measured according to SCAN-C 102 XE.

#### 2.2.2 Carbohydrate Content, Intrinsic Viscosity, and Molecular Mass Distribution

The pulp carbohydrate content was determined applying NREL/TP-510-42618 standard^29^ and Janson’s formula.^30^ The monosaccharides were analyzed by high-performance anion exchange chromatography with pulsed amperometric detection (HPAEC-PAD) in a Dionex ICS-3000 system, equipped with a CarboPac PA20 column.

The intrinsic viscosity ([η]) was measured according to standard SCAN-CM 15:88, while the molecular mass distributions were measured after derivatization with ethyl isocyanate as reported in the literature.^31^ The molecular mass distribution was determined in duplicate by size exclusion chromatography using a multiangle light scattering detector. The instrument included a Dionex Ultimate 3000 HPLC module, a Shodex DRI (RI-101) detector, and a Viscotek/Malvern SEC/MALS 20 multiangle light-scattering (MALS) detector. The column used was Agilent PLgel MIXED-A (×4). The flowrate and the injection volume were 0.75 mL/min and 100 μL, respectively.

#### 2.2.3 Supramolecular Structure

The cellulose crystallinity index and crystallite dimensions were measured as described elsewhere.^14^ XRD data were collected by a transmission mode of SmartLab instrument (RIGAKU, λ = 1.5418 Å). Crystallinity was estimated by the ratio of background and crystalline area after the background subtraction processes. The crystal size (CW_*hkl*_) was estimated using the Scherrer equation
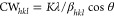
where *K* = 0.90 is the shape factor, λ is the X-ray wavelength, β_*hkl*_ is the full width at half-maximum of the diffraction peak in radians, and θ is the diffraction angle of the peak.

#### 2.2.4 Pulp Reactivity

Pulp reactivity was assessed using the dissolution-based torque reactivity (DTR) test.^32^ The DTR test evaluates the dissolution of an aqueous suspension of pulp fibers in cupriethylenediamine (CED) by monitoring the changes in torque. The dissolution is performed under standard conditions.^32^ Torque is plotted against time from the moment the solvent is injected until its stabilization in a plateau, which corresponds to the end of dissolution. The ease of dissolution is assessed by the dissolution rate, the dissolution time (DT), and the final torque. Pulps that dissolve faster are considered more reactive. Each sample was tested minimum in triplicate, and the average is reported.

## 3 Results and Discussion

### 3.1 Comparison Between Hardwood and Softwood Reference Pulps

**Figure 1:**
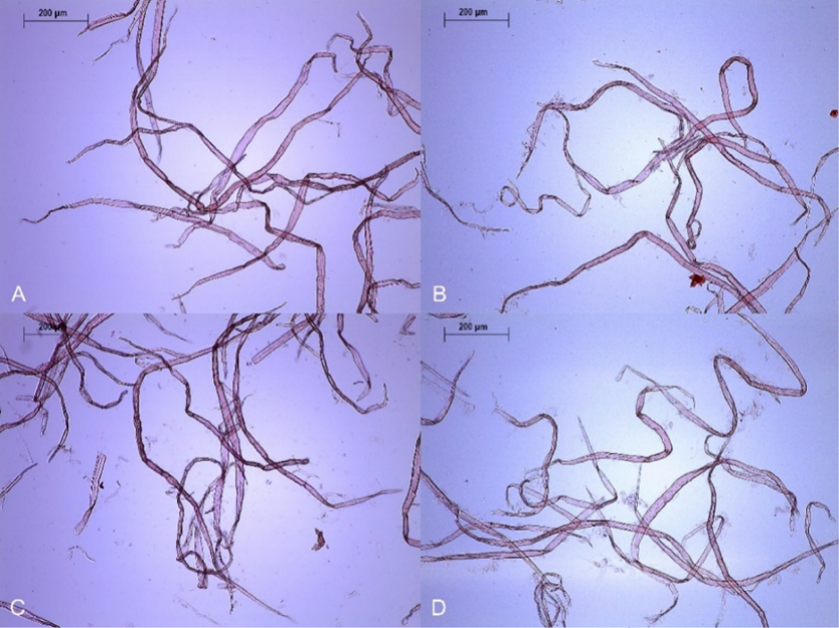
Hardwood samples under optical microscopy. (A) HW-PHK, (B) HW-E-120, (C) HW-XM-240, and (D) HW-EXM-240. All the treatments increased the amount of fines and the fibrillation extent.

**Figure 2:**
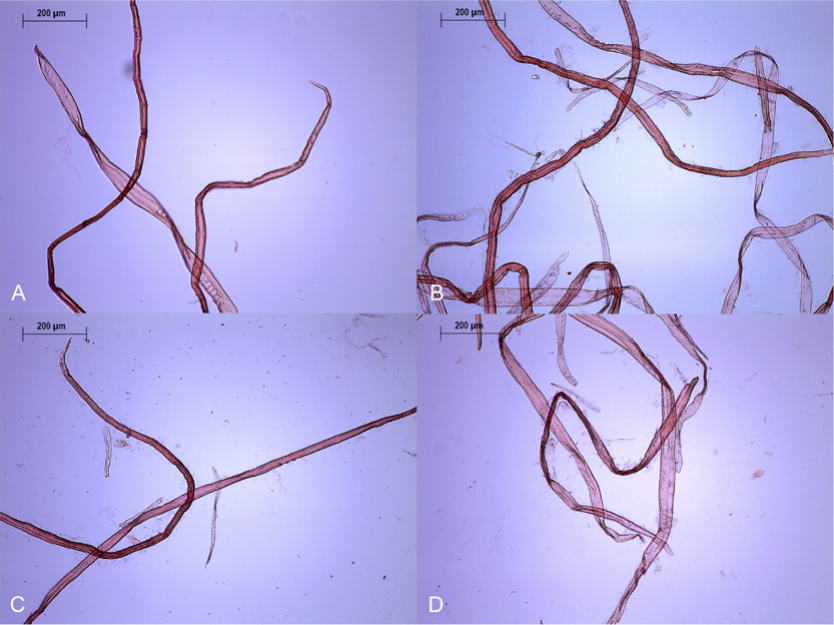
Softwood samples under optical microscopy. (A) SW-PHK, (B) SW-E-240, (C) SW-XM-240, and (D) SW-EXM-240. Fiber fibrillation increased with all treatments but particularly when endoglucanase, xylanase, and mannanase were used together.

**Figure 3:**
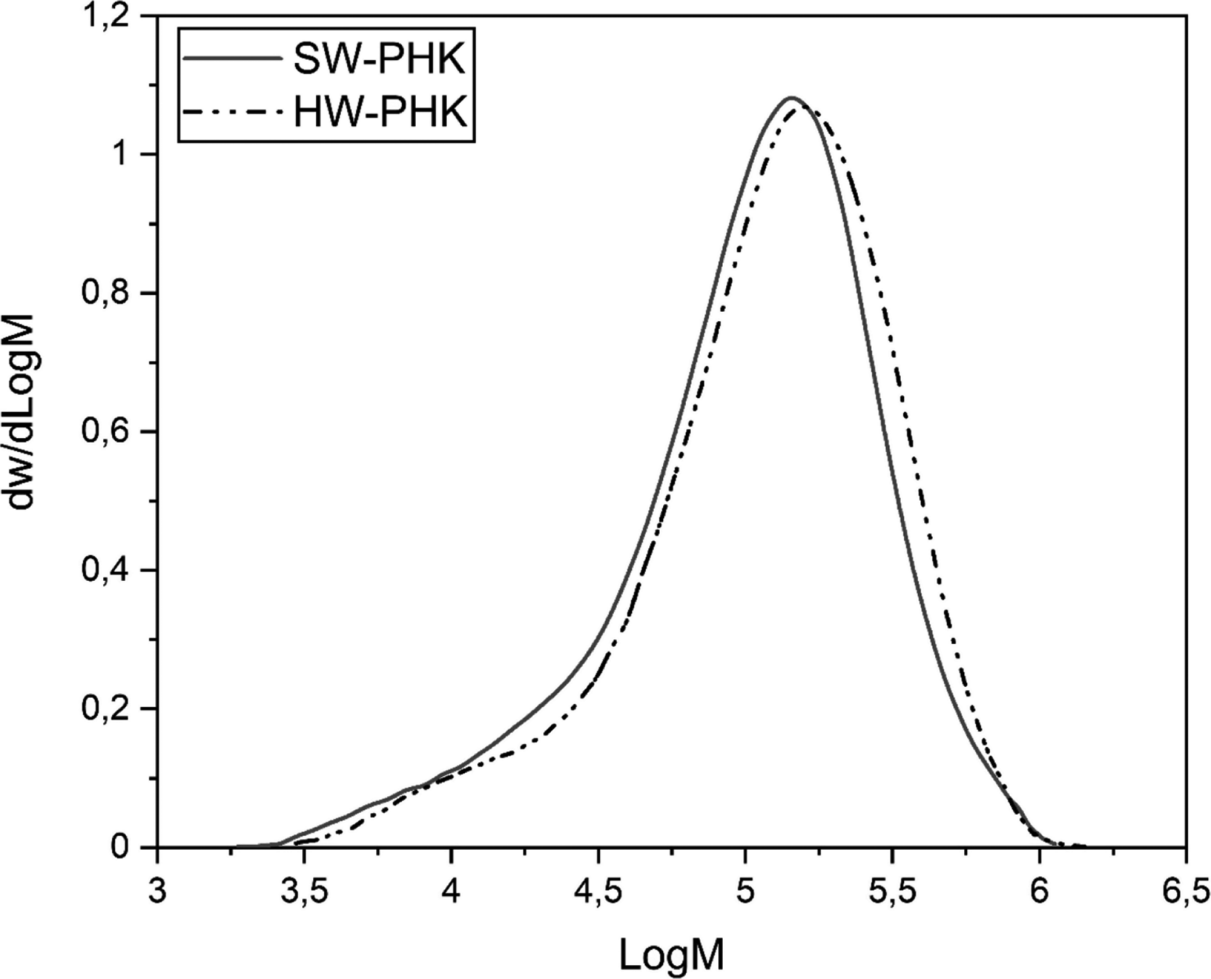
Molecular mass distribution of softwood (SW) and hardwood (HW) PHK reference pulps.

The mechano-enzymatic treatments of this study were performed on two prehydrolysis kraft pulps from hardwood and softwood species. As described in Tables 1 and 2, these pulps had different morphological, molecular, and supramolecular properties. The length of softwood fibers was more than twice that of hardwood fibers. Softwood cell wall thickness measured 7.09 ± 0.28 μm, while that of hardwood fibers was only 4.64 ± 0.14 μm. According to the fiber analyzer, softwood pulp had slightly more fines than hardwood pulp but much lower fibrillation. However, this difference in fibrillation was not as evident as in the optical microscope (Figures 1A and 2A). The overall porosity, represented by the FSP, was approximately the same for both the pulps, while the micropores of hardwood pulp were higher by ca. 0.15 mL/g. Similarly, the WRV of hardwood PHK was higher by 0.05 g/g. In terms of chemical composition, both pulps had a similar cellulose content (ca. 92–93%), but as predictable, they had different proportions of xylans and glucomannans. In softwood pulp, the content of xylans and glucomannans was almost the same, whereas in the hardwood pulp, xylans represented almost the whole hemicellulose fraction. As visible in Table 2 and Figure 3, the molecular mass distributions had similar number-average molecular weight (*M*_n_), weight-average molecular weight (*M*_w_), and polydispersity indices (PDIs). Nonetheless, hardwood pulp had a larger fraction of long-chain molecules. This was represented by the higher *Z* average molecular weight (*M*_z_), the larger percentage of molecules with degree of polymerization above 2000 (DP > 2000), and the higher intrinsic viscosity. Finally, at the supramolecular level, the crystallinity index and the crystallite dimensions of softwood pulp were higher than those of hardwood by 3.4% and 6 Å, respectively.

**Table 1:** Effect of the Mechano-Enzymatic Treatments on Fiber Morphology, Crystallinity Index (CI), and Crystallite Size (*L*_avg_)

	treatment	treatment time (min)	length (mm)	fines (%)	fibrillation (%)	FSP (mL/g)	micropores (mL/g)	CI (%)	*L*_avg_ (Å)	water retention value (g/g)
HW-PHK	ref.		0.78 ± 0.01	1.94 ± 0.13	3.72 ± 0.14	1.06 ± 0.00	0.53 ± 0.02	36.4 ± 0.1	32.3 ± 0.3	1.40 ± 0.02
	E	30	0.79 ± 0.01	2.10 ± 0.08	3.76 ± 0.02	1.04 ± 0.00	0.52 ± 0.01			1.31 ± 0.00
	E	60	0.79 ± 0.01	2.21 ± 0.20	3.99 ± 0.12	0.98 ± 0.02	0.45 ± 0.01	36.8 ± 0.2	33.3 ± 0.2	1.35 ± 0.01
	E	120	0.71 ± 0.00	4.20 ± 0.18	4.50 ± 0.32	0.96 ± 0.04	0.41 ± 0.03			1.36 ± 0.01
	XM	30	0.78 ± 0.01	2.01 ± 0.10	3.85 ± 0.01	0.81 ± 0.01	0.34 ± 0.01			1.29 ± 0.00
	XM	60	0.79 ± 0.01	2.16 ± 0.04	3.80 ± 0.08	0.85 ± 0.07	0.40 ± 0.01	36.9 ± 0.2	32.4 ± 0.1	1.29 ± 0.00
	XM	120	0.75 ± 0.01	2.72 ± 0.18	4.11 ± 0.29	0.83 ± 0.01	0.37 ± 0.00			1.25 ± 0.02
	XM	240	0.72 ± 0.01	3.30 ± 0.10	4.42 ± 0.17	0.83 ± 0.02	0.38 ± 0.02	39.4 ± 0.2	37.3 ± 0.3	1.24 ± 0.00
	EXM	30	0.78 ± 0.01	2.26 ± 0.02	3.93 ± 0.03	0.94 ± 0.01	0.38 ± 0.01			1.31 ± 0.00
	EXM	60	0.76 ± 0.00	2.73 ± 0.15	3.96 ± 0.08	1.00 ± 0.02	0.41 ± 0.00	37.2 ± 0.5	35.8 ± 3.6	1.34 ± 0.00
	EXM	120	0.75 ± 0.02	2.98 ± 0.44	4.11 ± 0.12	0.90 ± 0.00	0.38 ± 0.01			1.34 ± 0.00
	EXM	240	0.74 ± 0.01	3.18 ± 0.08	4.21 ± 0.32	0.96 ± 0.02	0.41 ± 0.03	37.5 ± 0.4	34.0 ± 0.5	1.41 ± 0.00
SW-PHK	ref.		1.97 ± 0.08	2.36 ± 0.23	1.39 ± 0.71	1.05 ± 0.00	0.38 ± 0.01	39.8 ± 0.1	38.3 ± 0.4	1.35 ± 0.01
	E	30	1.87 ± 0.02	2.89 ± 0.03	1.24 ± 0.09	1.08 ± 0.00	0.35 ± 0.00			1.37 ± 0.00
	E	60	1.85 ± 0.04	2.82 ± 0.24	1.43 ± 0.10	1.06 ± 0.01	0.35 ± 0.00	38.0 ± 0.2	35.3 ± 0.6	1.37 ± 0.01
	E	120	1.83 ± 0.05	2.87 ± 0.22	1.76 ± 0.21	1.04 ± 0.02	0.35 ± 0.03			1.39 ± 0.00
	E	240	1.77 ± 0.04	3.18 ± 0.13	1.96 ± 0.19	1.09 ± 0.02	0.35 ± 0.02	37.5 ± 0.1	35.2 ± 0.3	1.42 ± 0.00
	XM	30	1.86 ± 0.04	3.09 ± 0.25	2.04 ± 0.06	0.97 ± 0.01	0.34 ± 0.02			1.29 ± 0.01
	XM	60	1.85 ± 0.01	3.13 ± 0.42	2.27 ± 0.06	0.98 ± 0.03	0.33 ± 0.01	36.8 ± 0.3	33.7 ± 0.1	1.29 ± 0.00
	XM	120	1.90 ± 0.01	2.99 ± 0.21	2.30 ± 0.03	0.98 ± 0.01	0.33 ± 0.02			1.29 ± 0.01
	XM	240	1.81 ± 0.03	3.32 ± 0.30	2.27 ± 0.09	1.01 ± 0.01	0.35 ± 0.02	36.2 ± 0.2	33.2 ± 0.2	1.28 ± 0.01
	EXM	30	1.92 ± 0.06	2.77 ± 0.02	2.24 ± 0.10	1.05 ± 0.04	0.36 ± 0.01			1.34 ± 0.02
	EXM	60	1.83 ± 0.01	3.07 ± 0.21	2.16 ± 0.06	1.08 ± 0.02	0.38 ± 0.01	37.3 ± 0.2	33.9 ± 0.3	1.37 ± 0.01
	EXM	120	1.86 ± 0.01	2.80 ± 0.24	2.30 ± 0.01	1.04 ± 0.05	0.33 ± 0.01			1.37 ± 0.00
	EXM	240	1.84 ± 0.01	3.24 ± 0.25	2.44 ± 0.16	1.06 ± 0.01	0.36 ± 0.02	38.8 ± 0.1	37.1 ± 0.6	1.37 ± 0.03

**Table 2:** Effect of the Mechano-Enzymatic Treatments on Pulp Carbohydrates, Intrinsic Viscosity, and Molecular Mass Distribution

	treatment	treatment time (min)	cell (%)	Xyl. (%)	glucom. (%)	intrinsic viscosity (mL/g)	*M*_n_ (kDa)	*M*_w_ (kDa)	*M*_z_ (kDa)	PDI	DP < 200 (%)	DP > 2000 (%)
HW-PHK	ref.		92.8	6.4	0.8	475 ± 3	73 ± 3	185 ± 2	335 ± 9	2.5	4.2	16.4
	E	30	93.2	6.4	0.4	398 ± 1	50 ± 1	128 ± 5	241 ± 2	2.6	6.4	11.2
	E	60	92.6	7.0	0.4	382 ± 5	42 ± 4	132 ± 4	252 ± 9	3.1	8.3	10.7
	E	120	92.6	7.0	0.4	368 ± 1	46 ± 6	141 ± 12	262 ± 7	3.1	7.8	10.6
	XM	30	94.3	5.7	0.0	462 ± 12	77 ± 2	183 ± 1	317 ± 2	2.4	3.7	18.7
	XM	60	94.9	4.7	0.4	456 ± 10	74 ± 0	177 ± 1	321 ± 9	2.4	3.8	15.2
	XM	120	95.1	4.9	0.0	453 ± 11	74 ± 7	172 ± 4	301 ± 4	2.3	3.9	13.7
	XM	240	95.1	4.9	0.0	462 ± 6	74 ± 4	181 ± 1	319 ± 8	2.4	4.0	16.5
	EXM	30	95.1	4.9	0.0	394 ± 15	63 ± 9	160 ± 3	300 ± 12	2.6	5.0	12.6
	EXM	60	95.2	4.8	0.0	378 ± 5	62 ± 4	157 ± 1	298 ± 5	2.5	5.0	13.5
	EXM	120	95.1	4.9	0.0	371 ± 1	56 ± 7	151 ± 2	280 ± 7	2.7	6.0	13.1
	EXM	240	95.2	4.8	0.0	363 ± 0	53 ± 2	142 ± 1	280 ± 6	2.7	6.8	10.1
SW-PHK	ref.		93.8	2.6	3.6	463 ± 3	76 ± 0	178 ± 1	306 ± 7	2.3	3.6	15.2
	E	30	93.5	2.6	3.9	440 ± 11	61 ± 0	161 ± 0	288 ± 5	2.6	5.2	12.1
	E	60	93.4	2.6	4.0	428 ± 8	61 ± 0	159 ± 1	285 ± 2	2.6	5.3	12.4
	E	120	93.9	2.7	3.6	407 ± 5	63 ± 9	154 ± 3	270 ± 9	2.5	4.6	12.0
	E	240	94.2	2.5	3.3	378 ± 0	52 ± 3	148 ± 0	269 ± 6	2.8	4.5	11.0
	XM	30	93.6	2.5	3.9	454 ± 1	73 ± 13	170 ± 1	308 ± 4	2.4	2.8	11.7
	XM	60	93.5	2.5	4.0	443 ± 13	73 ± 2	175 ± 2	299 ± 2	2.4	3.8	14.8
	XM	120	93.7	2.4	3.8	455 ± 11	59 ± 12	172 ± 4	300 ± 4	3.0	5.5	15.5
	XM	240	93.5	2.7	3.8	450 ± 2	75 ± 2	173 ± 2	300 ± 6	2.3	5.6	8.7
	EXM	30	93.4	2.6	4.0	409 ± 2	70 ± 9	163 ± 2	287 ± 7	2.3	3.9	12.4
	EXM	60	94.1	2.4	3.5	407 ± 2	60 ± 6	158 ± 4	280 ± 10	2.6	5.3	12.4
	EXM	120	93.6	2.6	3.8	376 ± 5	62 ± 0	153 ± 1	270 ± 2	2.5	4.9	11.4
	EXM	240	93.8	2.5	3.7	377 ± 1	58 ± 1	150 ± 1	269 ± 3	2.6	5.4	11.1

**Figure 4:**
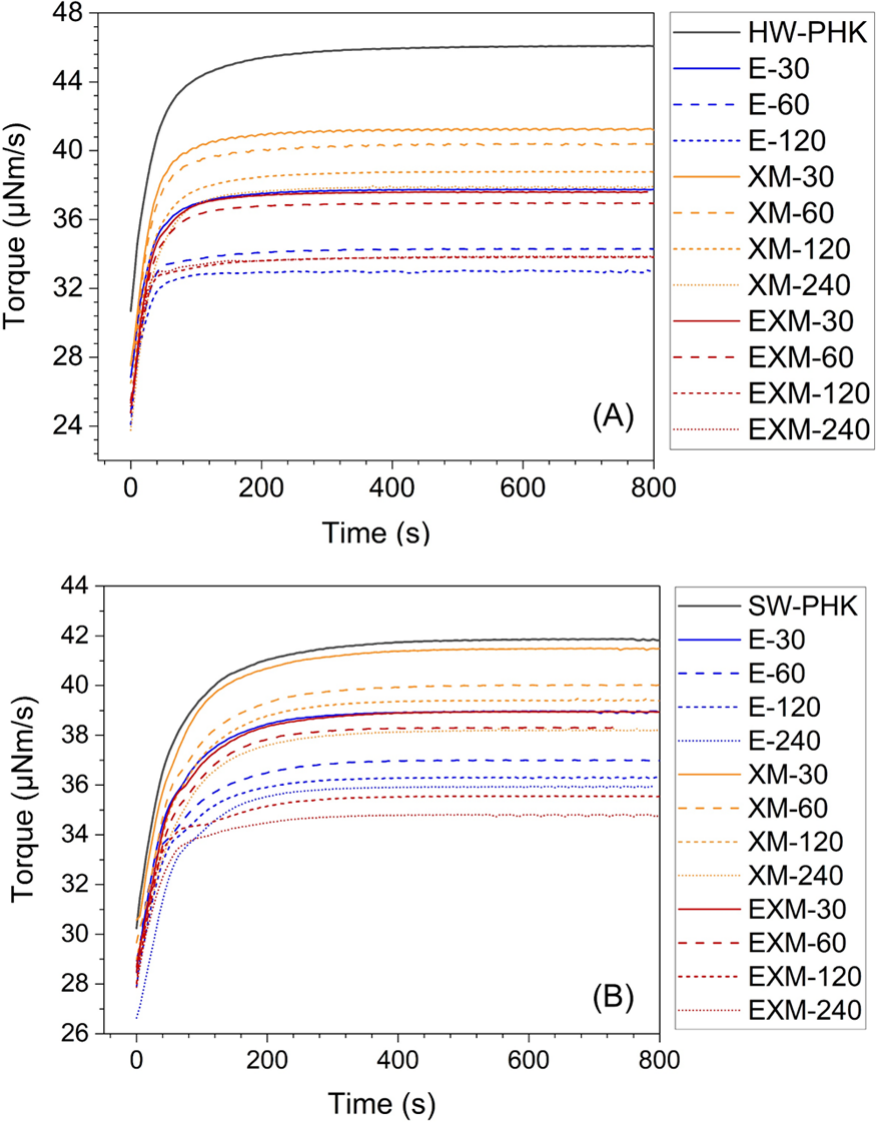
DTR dissolution curves of hardwood PHK pulp (A) and softwood PHK pulp (B). The color code refers to the treatments: endoglucanase treatments—blue, xylanase and mannanase treatments—orange, and endoglucanase, xylanase, and mannanase—red.

Because of the different morphology, chemical composition, and supramolecular organization, softwood and hardwood reference pulps had different dissolution times (Table 3, Figure 4). The hardwood dissolution time in CED was 32 s faster than the softwood dissolution time. It is reasonable that the dissolution time decreased when fines, fibrillation, and porosity were higher and crystallinity index and cellulose crystallite size were lower. Moreover, it is probable that the dissolution benefitted also by thinner cell walls.^33^

**Table 3:** Effect of the Mechano-Enzymatic Treatments on Pulp Dissolution

	treatment	treatment time (min)	dissolution time (s)	torque plateau (μNm)
HW-PHK	ref.		236 ± 32	45.48 ± 1.22
	E	30	148 ± 14	36.28 ± 1.38
	E	60	150 ± 5	34.45 ± 0.32
	E	120	87 ± 13	33.08 ± 0.21
	XM	30	168 ± 15	40.38 ± 0.77
	XM	60	163 ± 10	40.43 ± 0.12
	XM	120	152 ± 19	39.16 ± 0.45
	XM	240	148 ± 12	38.62 ± 0.71
	EXM	30	130 ± 17	37.62 ± 0.14
	EXM	60	133 ± 8	37.20 ± 0.33
	EXM	120	132 ± 14	34.62 ± 0.95
	EXM	240	141 ± 22	33.98 ± 0.34
SW-PHK	Ref.		268 ± 13	41.36 ± 0.42
	E	30	215 ± 11	39.43 ± 0.53
	E	60	211 ± 17	37.23 ± 0.53
	E	120	201 ± 17	36.80 ± 0.40
	E	240	188 ± 12	36.10 ± 0.49
	XM	30	256 ± 12	40.30 ± 1.15
	XM	60	230 ± 23	39.02 ± 1.12
	XM	120	241 ± 6	39.32 ± 0.50
	XM	240	226 ± 27	40.05 ± 0.20
	EXM	30	215 ± 14	38.17 ± 0.67
	EXM	60	216 ± 11	38.11 ± 0.25
	EXM	120	221 ± 16	36.63 ± 0.91
	EXM	240	188 ± 5	35.36 ± 0.43

### 3.2 Effect of Endoglucanase (E Treatments)

The endoglucanase-based mechano-enzymatic treatment of hardwood and softwood PHK pulps caused an extensive cellulose depolymerization and a modest change in fiber morphology.

The intrinsic viscosity of both hardwood and softwood pulps decreased with increasing treatment time, reaching a maximum viscosity reduction of ca. 23 and 18%, respectively (Table 2). As the potential application of the treated pulps could be the production of man-made cellulose fibers, extensive depolymerization was limited to avoid the deterioration of the mechanical properties of the end product. Therefore, the targeted intrinsic viscosity should have not exceeded 350 mL/g. Interestingly, compared to hardwood, softwood pulp required longer treatment times to reach the desired intrinsic viscosity.

In agreement with the intrinsic viscosity, the molecular mass distributions shifted toward shorter chain lengths. Both hardwood and softwood pulps showed the greatest shift within the first 30 min (Table 2). However, after this time, the depolymerization of the two pulps evolved differently. Longer treatments left the molecular weight averages of hardwood almost unchanged, but the fraction with DP > 2000 decreased and that with DP < 200 increased. In case of softwood, both the molecular weight averages and the fraction with DP > 2000 kept decreasing with the treatment time.

The effect of the treatment on hardwood and softwood fiber morphology was alike (Table 1). Hardwood and softwood fiber lengths shortened by maximum 9 and 10%, respectively. Consequently, there was a slight increase in the fines content. Both pulps became more fibrillated. However, as visible in Figures 1B and 2B, this fibrillation remained modest. Moreover, both pulps lost part of their initial porosity. Hardwood FSP and micropores decreased with increasing treatment time. After 120 min, porosity measured ca. 0.10 mL/g less than that before the treatment, and the change seems to be due principally to the closure of part of the micropores. In comparison, softwood micropores decreased less. In 30 min treatment, they reduced by 0.03 mL/g and then they did not decrease further. Softwood FSP showed a slight increase over the range of the experiment. It is possible to attribute the partial closure of the micropores to hornification, while the increase in FSP can be due to internal fibrillation. Both processes may be at work simultaneously, leading to complex changes in the fiber pore structure with some enzyme treatments.

Only signs of slight hornification were detected in XRD analysis (Table 1). The hardwood crystallinity index remained constant throughout the treatment, while its crystallite dimensions increased only slightly. Both softwood crystallite index and crystallite dimensions decreased. Because endoglucanase is known to preferentially degrade less-ordered cellulose,^34^ softwood results might appear unusual. A possible explanation is that the decrease in softwood crystallinity might not be due to the endoglucanase itself but rather due to the mechanical treatment. A previous study tested similar mixing conditions than those here in use, and it revealed that the mechanical action alone might cause a modest decrease in the crystallinity index.^14^

The WRV of softwood increased throughout the treatment, while the WRV of hardwood decreased within the first 30 min treatment and then increased again with longer treatment times. The initial decrease in hardwood WRV is too large to be ascribed to the reduced porosity. Perhaps, it resulted from the release of some fiber internal stresses. On the other hand, WRV increases were supported by the increase in fines and fibrillation. In case of softwood, the decrease in crystallinity and crystallite size may have contributed to the increase in fiber swelling.

All endoglucanase-based treatments shortened the original dissolution time of hardwood and softwood PHK pulps (Figure 4 and Table 3). The activation increased with the treatment time, and it was more remarkable in hardwood. After 30 min treatment, the dissolution time of hardwood and softwood decreased by 36 and 20%, respectively. No improvement occurred from 30 to 60 min. Thereafter, the dissolution time reduced further, and the shortest dissolution times were achieved at the longest treatment times. These corresponded to a total decrease by 63% for hardwood and by 30% for softwood.

**Figure 5:**
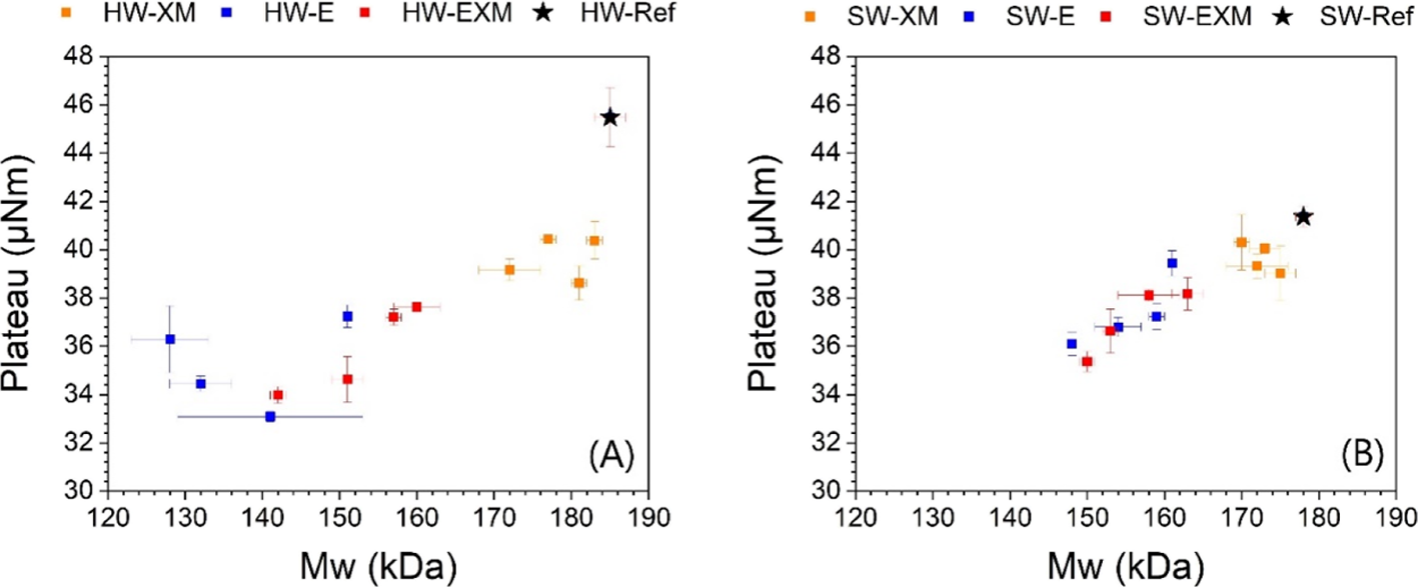
Correlation between plateau and weight-average molecular weight for hardwood pulps (A) and softwood pulps (B).

The dissolution time depends on several pulp features. In this set of samples, the dissolution was shorter when the intrinsic viscosity, the molecular weight averages, and the fraction of molecules with DP > 2000 were lower. Shorter chain molecules are faster to dissolve, and they contribute to the reduction of the final torque plateau of the dissolution curves (Figure 5). Moreover, the dissolution became faster with the release of fines and the increased fibrillation. In case of softwood pulps where the increase in fines and fibrillation was limited, it is plausible that the dissolution was favored by the decrease in crystallinity index and in crystallite size.

### 3.3 Effect of Combined Xylanase and Mannanase (XM Treatments)

The objective of the mechano-enzymatic treatment with xylanase and mannanase was to verify whether the selected hemicellulases could increase pulp purity and whether a decrease in the noncellulosic pulp components could have accelerated fiber direct dissolution. Hemicelluloses accounted only for 7.2 and 6.2% of the total carbohydrates of hardwood and softwood, respectively. Nonetheless, these residual xylans and glucomannans still reduce cellulose accessibility, especially when co-crystallized on cellulose with high molecular weight and reduced branching.

After the treatment, the intrinsic viscosity and the molecular weight lowered, proving that hemicelluloses were successfully depolymerized. Due to the limited hemicellulose contents, the changes were not drastic. Regardless of the wood species, the drop in intrinsic viscosity remained below 5%, while that of *M*_w_ and *M*_z_ below 10% (Table 2). The decrease occurred within the first 30 min, after which no significant changes occurred. Moreover, the depolymerization affected more *M*_w_ than *M*_z_, while *M*_n_ remained unchanged. This suggests that the depolymerization affected the fraction of hemicelluloses with higher molecular weight. It is reasonable that, acting on these long-chained hemicelluloses, the mechano-enzymatic treatment also weakened the bonding between hemicellulose and cellulose, therefore increasing the accessibility of the latter.

Interestingly, hemicellulose depolymerization reduced the hemicellulose content only in the case of hardwood pulp. Hardwood xylans decreased by a maximum of ca. 1.7%, whereas hardwood glucomannans were completely removed (Table 2). It is possible that softwood hemicelluloses were less accessible to xylanase and mannanase because of being differently distributed across the fiber cell wall or because softwood PHK had less micropores and higher crystallinity than hardwood PHK.

The XM treatments of softwood increased fibrillation and produced less fines than the XM treatments of hardwood perhaps because softwood pulp generally requires a greater refining energy input. Fines content and fibrillation increased with the treatment time, and they reached their maxima after 240 min. An example of hardwood and softwood fibers treated after 240 min is visible in Figures 1C and 2C.

The FSP and the micropores of hardwood pulp decreased by 20–24 and 25–36%, respectively, while those of softwood by 4–8 and 8–13% (Table 1). Thus, xylanase and mannanase reduced hardwood pulp porosity more than endoglucanase. This is possibly due to hornification phenomena enhanced by hemicellulose removal. Hemicelluloses are amorphous, high water binding polymers that promote cell wall swelling and can stabilize the fibrils, preventing fibril aggregation (hornification). Because hardwood hemicelluloses were degraded more than softwood hemicellulases, hardwood was more hornified. This is confirmed also by the WRV. Hardwood and softwood maximum decrease in the WRV that measured 0.16 and 0.07 g/g, respectively (Table 1). Possibly, this decrease was partly counteracted by the production of fines and fibrillation.

An additional sign that XM treatments hornified more hardwood than softwood pulp is visible in the increase in the hardwood crystallinity index and crystallite size with treatment time (Table 1). As with E treatments, the effect of XM treatments on softwood pulp was unexpected. The softwood crystallinity index and crystallite size decreased within the first 60 min and then remained stable. Perhaps, the decrease in crystallinity could be ascribed to the mechanical mixing^14^ or to a decrease in the fraction of hemicelluloses co-crystallized on cellulose.

It is interesting that lower porosity, crystallinity, and WRV did not lead to slower dissolutions. On the contrary, xylanase and mannanase significantly decreased the original pulp dissolution time without affecting the weight-average molecular mass as much as endoglucanase (Figure 4 and Table 3). The treatment was more effective on hardwood. After 30 min treatment, hardwood and softwood dissolution times decreased by 29 and 4%, respectively. The activation progressed with longer treatment times. After 240 min, the dissolution of hardwood and softwood pulps was shorter by 37 and 16%, respectively. It emerged that pulp dissolution time was affected more by hemicellulose depolymerization and by the increase in fines and fibrillation than by the decrease in porosity and swelling. Possibly, the dissolution was also promoted by a partial reorganization of xylan from twofold to threefold conformation. The flat twofold conformation has higher affinity for the cellulose surface than the threefold conformation, and it generates strong hydrogen bonding between xylan and cellulose.^35^ Falcoz-Vigne and coauthors propose that twofold adsorbed xylan molecules can get partially reorganized into threefold xylans, and this reorganization is promoted by the high flexibility of the chain ends and by shorter xylan chains.^35^ Considering this, xylan depolymerization could have contributed to loosening the interactions between xylans and cellulose.

### 3.4 Effect of Combined Endoglucanase, Xylanase, and Mannanase (EXM Treatments)

As discussed in Sections 3.2 and 3.3., mechano-enzymatic treatments with individual endoglucanase or mixed hemicellulases can reduce the dissolution time of dissolving pulps in CED by depolymerizing cellulose or high molecular weight xylans and glucomannans, respectively. Supported by the mechanical friction of mixing at high solid content, both treatments generate fines and fibrillation. Thus, it was considered whether a mechano-enzymatic treatment with all three enzymes combined could further decrease the dissolution time of hardwood and softwood PHK pulps.

According to *M*_w_ and *M*_z_, carbohydrate depolymerization was not enhanced using all the enzymes together (Table 2). Part of the decrease in intrinsic viscosity and molecular weight was due to hemicellulose depolymerization, which was more efficacious on hardwood pulp (Table 2). However, XM and EXM treatments reached the same final hemicellulose contents.

The changes in fiber length, fines content, and fibrillation induced by the EXM treatment were approximately the same as those measured after the treatment with endoglucanase alone (Table 1). For both wood species, longer treatment times slightly increased fines and fibrillation (Figures 1D and 2D).

EXM-treated hardwood samples had FSP and micropores in the same order of magnitude as E-treated and XM-treated samples, respectively (Table 1). The partial collapse of the micropores was attributed to hornification phenomena due to hemicellulose degradation, which were further confirmed by the increase of cellulose crystallinity and crystallite size. However, even if the EXM treatment hornified the pulp more than the endoglucanase alone, the two treatments led to similar WRV. Contrary to hardwood, EXM-treated softwood samples showed no meaningful changes in FSP or micropores (Table 1). Their WRV were, respectively, lower and higher than the WRV of E- and XM-treated softwood samples.

In summary, signs of synergy were visible only in the WRV results, while the fiber morphology, sugar analysis, molecular mass distribution, and DTR test showed no synergy (Table 3 and Figure 4). The dissolution times of EXM-treated pulps in CED were approximately in the same range as those achieved using endoglucanase alone. Only in the case of short treatments on hardwood, EXM treatments gave shorter dissolution times than E treatments. It is interesting to notice that hardwood treated by XM and EXM treatments for 240 min had a similar dissolution time, even if the sample treated with only xylanase and mannanase had lower porosity, higher crystallinity, lower water retention value, and higher molecular mass.

## 4 Conclusions

This study proved that mechano-enzymatic treatments with endoglucanase, xylanase, and mannanase can shorten the dissolution time of hardwood and softwood PHK pulps in CED. The dissolution was accelerated by both the enzymatic degradation of cellulose, xylans, and glucomannans and the mild refining action due to mixing at high pulp solid contents. Interestingly, the hemicellulases reduced the hemicellulose content of hardwood but not that of softwood. The main changes in *M*_w_ or intrinsic viscosity were registered already within the first 30 min. Longer treatment times could further extend the depolymerization but marginally. The efficiency of longer treatments was hampered by hornification phenomena. Hornification was more evident after hemicellulose degradation.

The maximum decrease in dissolution time was by 63 and 30% with endoglucanase and by 37 and 16% with xylanase and mannanase for hardwood and softwood, respectively. Xylanase and mannanase could significantly decrease the pulp dissolution time, while conserving a high molecular mass. This is useful, because the decrease of the molecular mass can weaken the mechanical properties of cellulose-based man-made fibers.^20^

At the studied conditions, the advantages of combining endoglucanase with xylanase and mannanase were negligible. Synergy was visible only in the WRV results. Whether endoglucanase was used alone or with the hemicellulases, the fines content, fibrillation, and depolymerization of the carbohydrates were approximately the same. The hemicellulose content decreased only in hardwood and to the same extent for both XM and EXM treatments. Finally, the dissolution times of EXM-treated hardwood and softwood decreased by a maximum of 45 and 30%, respectively.
